# Inhibition of Aldose Reductase Activates Hepatic Peroxisome Proliferator-Activated Receptor-**α** and Ameliorates Hepatosteatosis in Diabetic db/db Mice

**DOI:** 10.1155/2012/789730

**Published:** 2011-11-03

**Authors:** Longxin Qiu, Jianhui Lin, Fangui Xu, Yuehong Gao, Cuilin Zhang, Ying Liu, Yu Luo, James Y. Yang

**Affiliations:** ^1^State Key Laboratory for Stress Cellular Biology and Department of Biomedical Sciences, School of Life Sciences, Xiamen University, Xiamen 361005, China; ^2^School of Life Sciences, and Fujian Key Laboratory of Preventive Veterinary Medicine and Biotechnology, Longyan University, Longyan 364000, China; ^3^School of Nursing, The Third Military Medical University, Chongqing 400038, China; ^4^Xiamen University Laboratory Animal Center, Xiamen University, Xiamen 361005, China

## Abstract

We previously demonstrated in streptozotocin-induced diabetic mice that deficiency or inhibition of aldose reductase (AR) caused significant dephosphorylation of hepatic transcriptional factor PPAR**α**, leading to its activation and significant reductions in serum lipid levels. Herein, we report that inhibition of AR by zopolrestat or by a short-hairpin RNA (shRNA) against AR caused a significant reduction in serum and hepatic triglycerides levels in 10-week old diabetic db/db mice. Meanwhile, hyperglycemia-induced phosphorylation of hepatic ERK1/2 and PPAR**α** was significantly attenuated in db/db mice treated with zopolrestat or AR shRNA. Further, in comparison with the untreated db/db mice, the hepatic mRNA expression of *Aco* and *ApoA5*, two target genes for PPAR**α**, was increased by 93% (*P* < 0.05) and 73% (*P* < 0.05) in zopolrestat-treated mice, respectively. Together, these data indicate that inhibition of AR might lead to significant amelioration in hyperglycemia-induced dyslipidemia and nonalcoholic fatty liver disease.

## 1. Introduction

The polyol pathway is a glucose metabolic shunt that is defined by two enzymatic reactions catalyzed by aldose reductase (AR, AKR1B1, EC1.1.1.21) and sorbitol dehydrogenase (SDH, EC1.1.1.14), respectively [1, 2]. Biochemically, AR catalyzes the rate-limiting reduction of glucose to sorbitol, with the aid of cofactor NADPH. SDH converts sorbitol to fructose using NAD^+^. AR/the polyol pathway have been demonstrated to play important roles in the development and progression of diabetic complications in a number of tissues including kidney, retina, lens, and peripheral neuron tissues [3–5]. In the liver, however, the expression of AR is relatively low under normal physiological conditions [6, 7]. By contrast, the hepatic expression of sorbitol dehydrogenase, the second enzyme for the polyol pathway, is quite high [8]. Due to the relatively lower levels of expression of AR in the liver under normal situations, relatively little attention had been paid to its roles in the liver in the past. Recently, however, increasing evidence has suggested that hepatic AR is dynamically regulated under a variety of conditions. For instance, in rats fed with fructose, hepatic AR is significantly upregulated, which is associated with impaired activation of Stat3 and suppressed activity of PPAR*α* in the liver [9]. In the Long Evans Cinnamon rats, induction of hepatic AR expression was shown to be associated with the development of hepatitis and hepatoma [10]. Similarly, significant upregulation of AR has also been demonstrated in other diseased liver tissues from rodents to humans [11–13].

The liver tissue plays a major role in energy metabolism, particularly glucose and lipid homeostasis. It is known that diabetes, type II diabetes mellitus (T2DM) in particular, is often associated with hepatic accumulation of triglycerides in both rodents and humans, which might eventually lead to the development of hepatic steatosis or nonalcoholic fatty liver disease (NAFLD) [14–16]. Recently, we demonstrated that deficiency or inhibition of AR caused significant dephosphorylation of hepatic PPAR*α*, leading to the activation of this transcriptional factor as well significant reduction in serum TG levels in streptozotocin-(STZ-) diabetic mice, an experimental model for type I diabetes mellitus (T1DM) [17]. Because T2DM is clinically much predominant than T1DM, in this current study, we wanted to determine whether AR also affects PPAR*α* in the liver of T2DM db/db mouse models. Furthermore, we wanted to determine how changes in AR activity might affect the hepatic lipid accumulation in the db/db mice. Our data suggest that inhibition of AR in the T2DM db/db mice led to significant activation in hepatic PPAR*α* and significant reductions in serum triglycerides (TG) and hepatic TG, suggesting that under hyperglycemia, AR/the polyol pathway might be greatly upregulated to contribute significantly to the hepatic regulation of TG metabolism and the development of nonalcoholic steatohepatitis (NASH) or nonalcoholic fatty liver disease (NAFLD).

## 2. Materials and Methods

### 2.1. Antibodies and Reagents

Antibodies were obtained from the following vendors, respectively: ERK1/2 and phospho-ERK1/2 (#9100), Cell Signaling (Beverly, Mass); PPAR*α* (sc9000) and AR (sc17735), Santa Cruz Biotechnology Inc. (Santa Cruz, Calif); phosphoserine-12 PPAR*α* (ab3484) and phosphoserine-21 PPAR*α* (ab3485), Abcam (Cambridge, UK); *β*-actin (A1978), Sigma (St. Louis, Mo). Oil-red O and other reagents were of analytical grade quality and from Sigma (St. Louis, Mo). Zopolrestat (zopol) was synthesized by the Department of Medicine Chemistry, Pfizer Global Research and Development (Groton, Conn).

### 2.2. Lentivirus shRNA Construct

Recombinant lentiviral vector expressing small hairpin RNA (shRNA) against mouse *AR* (pLV-shAR) and its control (pLV-shNC) were constructed by inserting double-strand shRNA oligonucleotides into plasmid pLentiLox3.7 (pLL3.7) at the *Hap*I and *Xho*I sites. Control and shRNA oligonucleotides against mouse AR were designed according to Ambion guidelines, with the sequences being 5′-ctggtcacacaacagaga-3′ and 5′-tacctaactcaggagaag-3′, respectively. Preparations of lentiviruses were performed by cotransfecting the lentiviral constructs with the packaging vectors into 293T cells using Lipofectamine 2000 (Invitrogen). Virus-containing supernatants were collected 48 h after infection. Viruses were recovered by ultracentrifugation at 110,000 ×g for 1.5 h and resuspended in PBS. Titers were determined by infecting 293T cells with serial dilutions of concentrated lentiviral preparations.

### 2.3. Animal Experiments

The animal experiments were conducted according to protocols and guidelines approved by the Xiamen University Institutional Animal Care and Use Committee. The db/m (BKS.Cg-m/Lepr^db^/J) mice were obtained from the Jackson Laboratory (Bar Harbor, Maine) and bred to obtain six-week-old male db/db mice and their lean control db/m mice for this study. All animals were maintained on standard laboratory chow under a 12 : 12 h light-dark schedule. For AR inhibition by zopolrestat (zopol) treatment, six-week-old db/db mice were randomly divided into four experimental groups, namely, db/m mice, db/m mice + zopol, db/db mice, and db/db mice + zopol. For zopol treatments, the mice were administrated with 50 mg/kg body weight/day of zopol as a single daily intraperitoneal injection for 28 days. The same volumes of saline were also administrated to other control groups of mice. For *in vivo* AR knock-down experiments, six-week-old db/db mice were randomly grouped (4 mice/group). *In vivo* transduction of lentiviruses was achieved through tail vein injections of 0.1 mL of concentrated viral suspension with a viral titer of 1.0 × 10^9^ IFU/mL in PBS. Twenty-eight days after zopol treatment or lentiviral injection, mice were sacrificed and tissues were dissected and immediately frozen in liquid N_2_ and stored at −80°C until use.

### 2.4. Semiquantitative Analyses of mRNA Expression by RT-PCR

Total RNA was isolated from tissues using Trizol Reagent (Invitrogen) according to the manufacturer's protocol. RT-PCR was performed to determine the levels of acetyl CoA oxidase (*Aco*), carnitine palmitoyl transferase-1 (*Cpt1*), apolipoprotein C-III (*ApoC3*), and apolipoprotein A-V (*ApoA5*) mRNAs as previously described [17]. The primers used were 5′-CCGCCACCTTCAATCCAGAGTTA-3′ and 5′-TCACAGTTGGGCTGTTGAGAATG-3′ (*Aco*), 5′-GGACGAATCGGAACAGGGATA-3′ and 5′-CCTTGTAATGTGCGAGCTGCA-3′ (*Cpt1*), 5′-CCTCTTGGCTCTCCTGGCATCT-3′ and 5′-TGCTCCAGTAGCCTTTCAGGG-3′ (*ApoC3*), 5′-GTGGGAGAAGACAC-CAAG-GCTC-3′ and 5′-GGTCAATGGCCTGAGTAAA-TGC-3′ (*ApoA5*), 5′-CGAGACCCCACTAA-CATCAAA-3′ and 5′-AGTCTTCTGGGTGGCA-GTGAT-3′ (GAPDH). DNA amplification was carried out using a High-Fidelity PrimeScript RT-PCR Kit (TaKaRa). The PCR products were electrophoresed on 2% agarose gels and visualized by staining with ethidium bromide. The integrated density values of the bands representing amplified products were acquired and analyzed by Image-Pro Plus software (Media Cybernetics, USA). 

### 2.5. Western Blot Analyses

Tissues were homogenized with Polytron in ice-cold buffer (1% Triton X-100, 50 mM HEPES pH 7.5, 150 mM NaCl, 1 mM EDTA, 10% glycerine, 10 mM Na_4_P_2_O_7_, 20 mM glycerophosphate, 10 mM NaF, 10 mM sodium orthovanadate, and proteinase inhibitor mixture). The protein concentrations of the extracts were measured using a bicinchoninic acid protein assay kit (Pierce). 40 *μ*g protein of each sample was loaded and separated on a 12% SDS-polyacrylamide gel and transferred to polyvinylidene difluoride (PVDF) membranes (Millipore). Blotted membranes were then incubated either anti-ERK or anti-phospho-ERK (1 : 1000) or anti-PPAR*α* (1 : 500) or anti-phospho-PPAR*α* (1 : 1000) or anti-AR (1 : 500) in TBS-0.1% Tween-20 with 5% nonfat milk at 4°C overnight. After several washes, the membranes were incubated with horseradish peroxidase-conjugated anti-rabbit IgG or anti-goat IgG (1 : 2000) in TBS-0.1% Tween-20 with 5% nonfat milk. The detection was achieved using the supersignal chemiluminescent substrate kit (Pierce). 

### 2.6. Blood Sample Analyses

Serum TG levels were measured using a colorimetric assay (Sigma, TR0100). Total serum cholesterol was measured using a cholesterol reagent kit (Jiancheng Biotech, Nanjing, China).

### 2.7. Liver TG Analyses

Liver TG was extracted by chloroform/methanol. Briefly, pulverized liver was homogenized in PBS, then extracted with chloroform/methanol (2 : 1), dried overnight, and resuspended in a solution of 60% butanol 40% Triton X-114/methanol (2 : 1). Liver total TG levels were measured using a colorimetric assay (Sigma, TR0100).

### 2.8. Oil-Red O Staining

Frozen liver sections of 10 *μ*m thickness were fixed in 4% paraformaldehyde and stained with 0.5% oil-red O using standard procedures.

### 2.9. Statistical Analyses

All data were processed and analyzed by GraphPad software (Prism 5.0) and expressed as mean ± SEM. Students' *t*-test was used for pair-wise comparisons and one-way ANOVA with Bonferroni's Multiple Comparison Test for multigroup analyses. Probability values less than 0.05 (*) were considered to be statistically significant; those less than 0.01 (**) more so.

## 3. Results

### 3.1. AR Inhibition-Reduced Serum TG but Not Serum TC Levels in Diabetic db/db Mice

**Figure 1:**
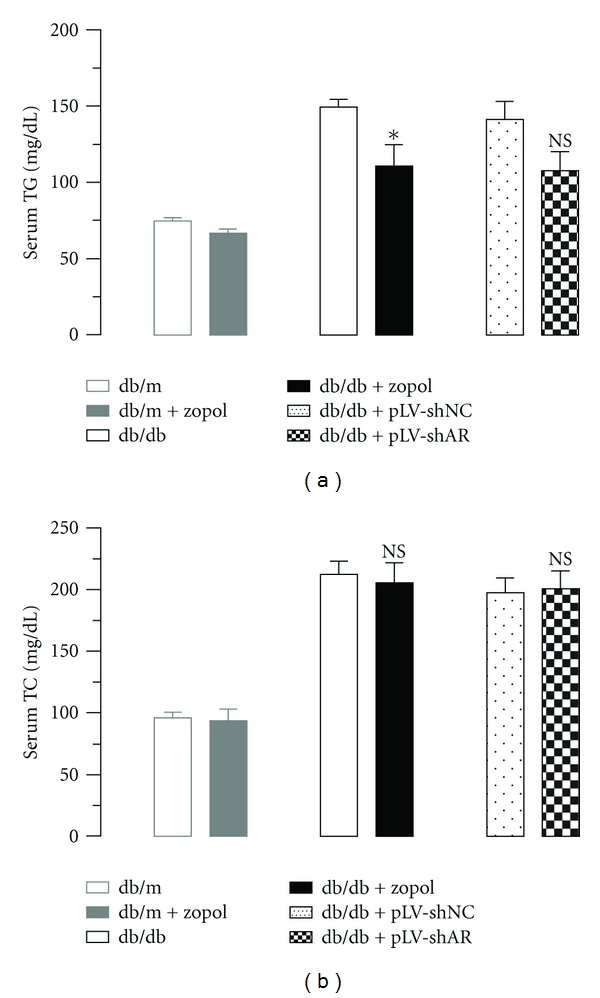
Effect of zopol treatment or AR knock-down on serum TG levels (a) and TC levels (b) of db/db mice. Lean control mouse groups are db/m, *n* = 6 and db/m + zopol, *n* = 4; diabetic mouse groups are db/db, *n* = 6; db/db + zopol, *n* = 6 and db/db + pLV-shNC (*n* = 4); db/db + pLV-shAR (*n* = 4). Values are expressed as the mean ± SEM. *P < 0.05; NS: not significant.

To determine the effects of AR on systemic lipid metabolism, we measured the serum TG and TC levels in db/db mice after zopol treatment or AR knockdown (Figure 1). As shown in Figure 1(a), zopol treatment for 4 weeks caused a significant reduction in the serum TG levels in the 10-week-old male db/db mice (110.6 ± 14.17 mg/dL for db/db + zopol versus 149.3 ± 5.06 mg/dL for db/db, *P* < 0.05) but had little effects on the control db/m mice. A similar reduction in serum TG level was also observed in 10-week-old db/db mice transduced with lentiviruses carrying shRNA for AR (107.6 ± 12.38 mg/dL for db/db + pLV-shAR versus 141.6 ± 11.51 mg/dL for db/db + pLV-shNC, *P* > 0.05), although the difference was not significant statistically. In contrast to serum TG, no significant change in serum TC levels was observed in both db/db mice treated with zopol or db/db mice transduced with lentiviruses carrying AR shRNA (Figure 1(b)), which is consistent with our previous findings in the STZ-induced T1DM mouse model [17]. Together these results indicate that inhibition of AR leads to significant reductions in serum TG but not serum TC. 

### 3.2. AR Inhibition-Reduced Hepatic TG in Diabetic db/db Mice

**Figure 2:**
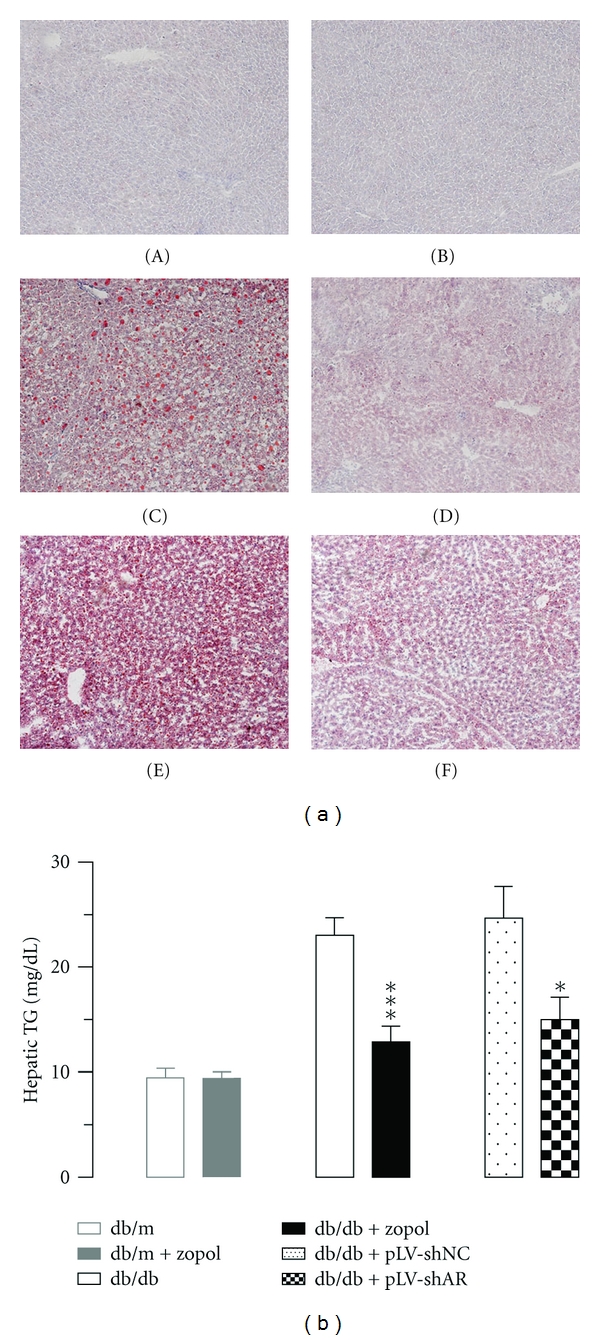
Effect of zopol treatment or AR knockdown on hepatic lipid in db/db mice. (a) Oil-red O staining of liver tissues of db/db mice after zopol treatment. (A) db/m; (B) db/m + zopol; (C) db/db; (D) db/db + zopol; (E) db/db + pLV-shNC; (F) db/db + pLV-shAR. Results are typical for 3 mice/group. Original magnification, ×100. (b) AR inhibition or AR knock-down reduced liver TG of db/db mice as analyzed chemically. Lean control mouse groups are db/m (*n* = 6) and db/m + zopol (*n* = 4); diabetic mouse groups are db/db (*n* = 6); db/db + zopol (*n* = 6) and db/db + pLV-shNC (*n* = 4); db/db + pLV-shAR (*n* = 4). Values are expressed as the mean ± SEM. *P < 0.05; ***P < 0.001.

To determine how changes in AR expression and activity might affect hepatic lipid accumulation, we analyzed the TG contents in the liver tissues of 10-week-old male db/db mice after zopol treatment for 28 days or lentivirus-mediated AR knockdown. Oil-red O staining of liver tissues showed that substantial fat droplets were diffusely distributed in the hepatic lobules from the 10-week-old untreated db/db mice. In the liver of the db/db mice received zopol for 28 days, however, much little and smaller fat droplets were observed (Figure 2(a)). Similar reductions in hepatic lipid accumulation were observed in db/db mice treated with lentiviruses containing AR-shRNA expression cassette. The results from tissue staining were further confirmed by biochemical analyses of hepatic TG content (Figure 2(b)). Zopol treatment in db/db mice significantly reduced hepatic TG by about 60% (12.89 ± 1.47 mg/g tissue for db/db + zopol versus 23.06 ± 1.66 mg/g tissue for db/db, *P* < 0.001). Similarly, AR knockdown in db/db mice also significantly reduced hepatic TG by about 40% (14.99 ± 2.11 mg/g tissue for db/db + pLV-shAR versus 24.69 ± 3.02 mg/g tissue for db/db + pLV-shNC, *P* < 0.05). 

### 3.3. AR Inhibition Led to Significant Dephosphorylation of Hepatic ERK1/2 and PPAR*α* in the db/db Mice

**Figure 3:**
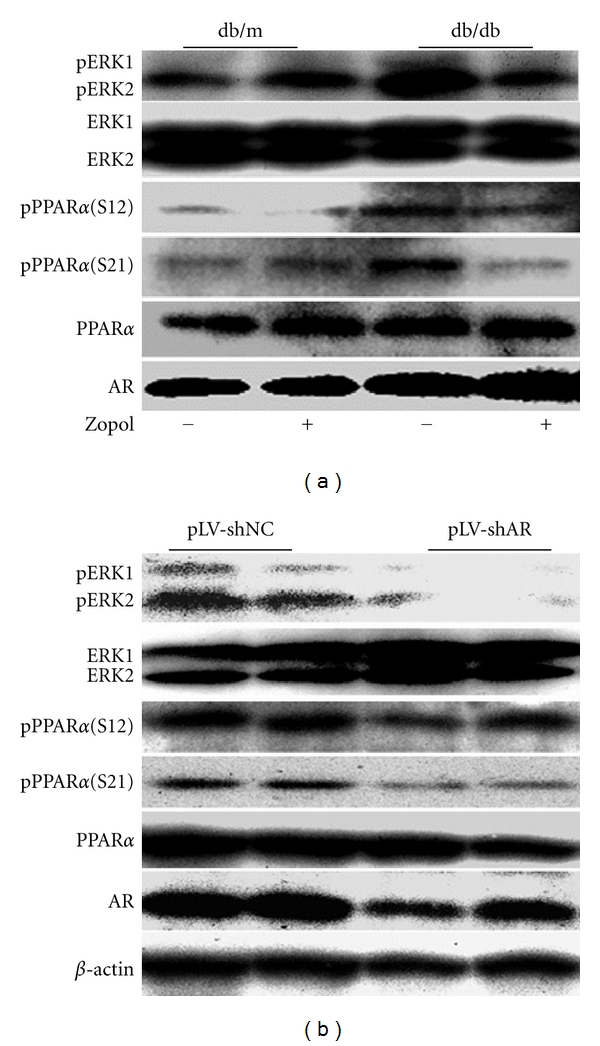
Effects of AR on PPAR*α* and ERK1/2 phosphorylation in db/db mice. (a) Representative Western blot for four independent experiments. Liver tissues were dissected and analyzed 28 days after zopol treatment. (b) Representative Western blot for four independent experiments. Liver tissues were dissected and analyzed 28 days after transduction with lentiviruses containing pLV-shAR or pLV-shNC. pERK1/2, phospho-ERK1/2; pPPAR*α* (S12), phosphoserine-12 PPAR*α*; pPPAR*α* (S21), phosphoserine-21 PPAR*α*.

In STZ-induced T1DM mice, we demonstrated previously that AR deficiency or AR inhibition led to significant dephosphorylation of hepatic ERK1/2 and PPAR*α* [17]. To determine whether this is also the case in the T2DM db/db mice, we examined the hepatic expression and phosphorylation of these proteins in 10-week-old db/db mice and its control db/m mice. As shown in Figure 3(a), with the elevation in hepatic AR expression in the db/db mice, the phosphorylation of both ERK1/2 and PPAR*α* (at Serine-12 and Serine-21) in the db/db mice was significantly enhanced. In db/db mice received zopol treatment for 28 days, however, the phosphorylation of both ERK1/2 and PPAR*α* was greatly attenuated. Noteworthy is that a slight increase in phosphoserine-21 PPAR*α* level was also observed for db/m mice following the zopol treatment. However, statistical analyses indicated the increase in pPPAR*α* (S21) in db/m mice with zopol is not significant (data not shown). Similar attenuations in phosphorylation of both ERK1/2 and PPAR*α* were also observed for db/db mice transduced with lentiviruses carrying shRNA for AR (Figure 3(b)). Together these results suggest that, consistent with the results from the STZ-induced T1DM mice, *in vivo* inhibition of AR in T2DM db/db mice also lead to dephosphorylation of both ERK1/2 and PPAR*α*, which might eventually lead to the activation of hepatic PPAR*α* to significantly affect hepatic lipid metabolism.

### 3.4. Dephosphorylation of PPAR*α* Is Associated with Altered Expression of Hepatic *Aco* and *ApoA5*

**Figure 4:**
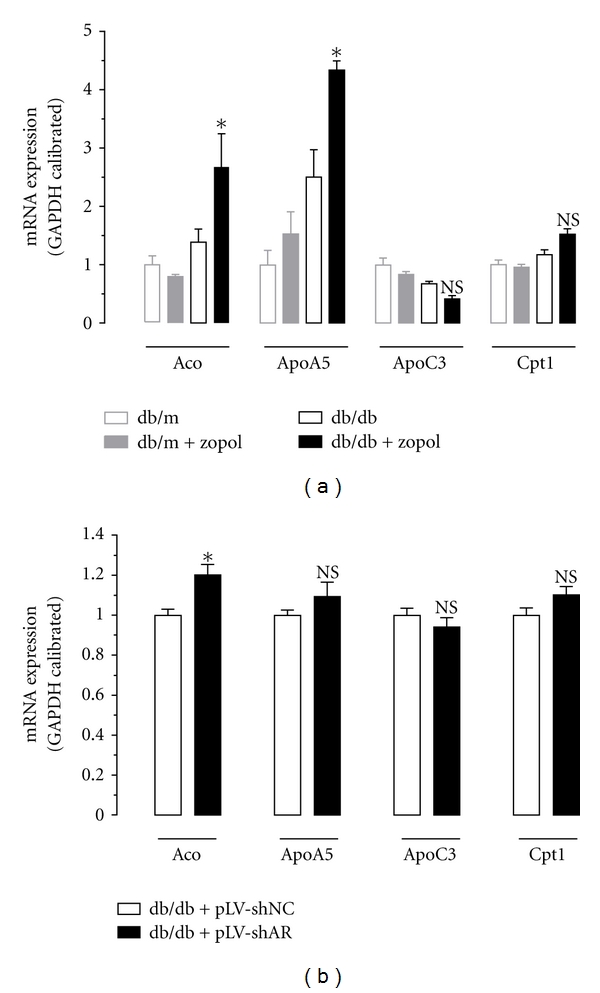
Hepatic mRNA expression of *Aco*, *ApoA5*, *ApoC3,* and *Cpt-1* as analyzed by semiquantitative RT-PCR in db/db mice. (a) Liver tissues were dissected and analyzed 28 days after zopol treatment. (b) Liver tissues were dissected and analyzed 28 days after transduction with lentiviruses containing pLV-shAR or pLV-shNC. Values are expressed as the mean ± SEM (*n* = 3-4). * *P* < 0.05; NS: not significant.

To determine the effects of PPAR*α* dephosphorylation on its target genes, we analyzed the expression of hepatic *Aco*, *ApoA5*, *ApoC3,* and *Cpt-1* mRNAs by semiquantitative RT-PCR for liver tissues from the control mice and db/db mice received zopol treatment or db/db mice transduced with lentiviruses carrying AR shRNA. As shown in Figure 4(a), compared with untreated db/db mice, mRNA expression of *Aco* and *ApoA5* in zopol-treated db/db mice elevated by approximately 93% (*P* < 0.05) and 73% (*P* < 0.05), respectively. Meanwhile, zopol-treated db/db mice had a slight but not significant lower hepatic expression of *ApoC3* mRNA expression and a slight but not significant higher expression of *Cpt-1* mRNA expression than the untreated db/db mice. The upregulation of hepatic *Aco* mRNA after zopol treatment was further confirmed in db/db mice transduced with lentiviruses carrying AR shRNA (Figure 4(b)). Probably due to incomplete knockdown, however, no significant changes were observed for hepatic mRNA expression of *ApoA5*, *ApoC3*, and *Cpt-1*. Together these data indicate that inhibition of AR caused activation of hepatic PPAR*α* to alter the expression and activity of major hepatic enzymes involved in lipid homeostasis in the T2DM db/db mice, which might have significant impact on hepatic lipid accumulation and the development or progression of NASH and NAFLD.

## 4. Discussion

AR/the polyol pathway is widely recognized to be involved in the pathogenesis of diabetic complications such as cataracts, nephropathy, and neuropathy [4, 18]. In contrast, relatively little attention has been paid to their potential roles in the development of diabetic lipid disorders. In spite of this, several studies have shown the possible link between activation/deactivation of AR/the polyol pathway and altered regulation in lipid metabolism. It has been reported that in diabetic patients with dyslipidemia, there are significant increases in plasma or serum and urinary sorbitol and fructose, indicating that the increased flux in the polyol pathway is concomitant with diabetic dyslipidemia [19, 20]. Moreover, pharmacological administration of several AR inhibitors including zopol were shown to reduce blood TG in rats [21], tumor bearing mice [22], and diabetic human patients [23], respectively. More recently, our group reported that in STZ-induced T1DM mouse models, genetic AR deficiency or *in vivo* inhibition by chemical inhibitors of AR significantly improved hyperglycemia-induced dyslipidemia [17]. It is therefore of interest to determine whether AR regulates PPAR*α* and affects hepatic lipid metabolism in T2DM models. In line with our expectation, we demonstrated in this current study that inhibition of AR by zopol treatment or transduction with lentiviruses carrying shRNA for AR greatly reduced hyperglycemia-induced lipid accumulation and hepatic steatosis in T2DM db/db mice. Furthermore, we showed that AR probably regulates hepatic lipid metabolism in part by modulating the status of PPAR*α* phosphorylation to alter its activity.

 In our current study, we utilized both chemical inhibitor and mRNA knockdown as means for the inhibition of AR. The inclusion of AR knockdown as an alternative approach for the inhibition of AR was necessary because we wanted to exclude the possible side or toxic effects and nonspecific inhibition that might be associated with chemical inhibitors of AR. Although both chemical inhibition and AR knock-down resulted in similar effects on hepatic lipid metabolism and mRNA expression levels of PPAR*α* and its target genes, AR knockdown appeared to be slightly less effective than zopol treatment. This is probably due to incomplete knockdown of AR as only a single injection was performed and that was maintained for 4 weeks before the analyses. 

The exact mechanisms underlying suppression of lipid accumulation or hepatic steatosis by inhibition of AR are not completely clear at this moment and require further investigations. At this moment, however, the mechanisms for increased lipid degradation and mechanisms for decreased lipid synthesis both appear to be functional. Our demonstration that inhibition of AR led to the activation of PPAR*α* through its dephosphorylation contributes in part to increased hepatic lipid degradation following inhibition of AR. It is well established that PPAR*α* is a central regulator for hepatic glucose and lipid metabolism as well as the development of lipid disorders including hepatic steatosis and NAFLD [24–29]. Once activated, it will tend to promote lipid catabolism by upregulating the expression of lipid catabolic enzymes such as lipoprotein lipase and *ApoA5* [30] and downregulating *ApoC3* [31]. Consistent with this, two important lipid catabolic enzymes *Aco* and *ApoA5* were significantly upregulated as a consequence of PPAR*α* activation, although not much change in mRNA expression was observed for *Cpt1* and *ApoC3*. Inhibition of AR, on the other hand, might also result in reduced lipid synthesis. Under hyperglycemic conditions, for example, abundant glucose might be channeled into the hyperglycemia-activated AR/the polyol pathway to generate a substantial amount of fructose in the liver. Fructose has long been known to be highly lipogenic and can contribute significantly to hepatic lipogenesis, adipogenesis, insulin resistance, obesity, hypertension, metabolic syndrome, hepatic steatosis, and NAFLD [9, 32–47] in both human and rodents. When AR is inhibited or when the polyol pathway is blocked, it can therefore be expected that endogenous hepatic fructose generation will be greatly reduced such that fructose-induced lipogenesis in the liver will also be suppressed, thereby leading to the suppression of hepatic steatosis or NAFLD. 

## Abbreviations

*Aco*: Acetyl CoA oxidase
*ApoA5*: Apolipoprotein A-V
*ApoC3*: Apolipoprotein C-III
AR: Aldose reductase
*Cpt1*: Carnitine palmitoyl transferase-1
ERK: Extracellular signal-regulated kinase
NAFLD: Nonalcoholic fatty liver disease
NASH: Nonalcoholic steatohepatitis
PPAR*α*: Peroxisome proliferator-activated receptor *α*
RT-PCR: Reverse transcription polymerase chain reaction
shRNA: Short-hairpin RNA
TG: Triglyceride
T1DM: Type I diabetes mellitus
T2DM: Type II diabetes mellitus
zopol: Zopolrestat.
